# Modeling-Enabled Characterization of Novel NLRX1 Ligands

**DOI:** 10.1371/journal.pone.0145420

**Published:** 2015-12-29

**Authors:** Pinyi Lu, Raquel Hontecillas, Vida Abedi, Shiv Kale, Andrew Leber, Chase Heltzel, Mark Langowski, Victoria Godfrey, Casandra Philipson, Nuria Tubau-Juni, Adria Carbo, Stephen Girardin, Aykut Uren, Josep Bassaganya-Riera

**Affiliations:** 1 The Center for Modeling Immunity to Enteric Pathogens, Virginia Bioinformatics Institute, Virginia Tech, Blacksburg, Virginia, 24061, United States of America; 2 Nutritional Immunology and Molecular Medicine Laboratory (www.nimml.org), Virginia Bioinformatics Institute, Virginia Tech, Blacksburg, Virginia, 24061, United States of America; 3 BioTherapeutics, 1800 Kraft Drive, Suite 200, Blacksburg, Virginia, 24060, United States of America; 4 Laboratory of Medicine & Pathobiology, University of Toronto, Toronto, Ontario, Canada; 5 Georgetown University Medical Center, Washington, District of Columbia, 20057, United States of America; University of Iowa Carver College of Medicine, UNITED STATES

## Abstract

Nucleotide-binding domain and leucine-rich repeat containing (NLR) family are intracellular sentinels of cytosolic homeostasis that orchestrate immune and inflammatory responses in infectious and immune-mediated diseases. NLRX1 is a mitochondrial-associated NOD-like receptor involved in the modulation of immune and metabolic responses. This study utilizes molecular docking approaches to investigate the structure of NLRX1 and experimentally assesses binding to naturally occurring compounds from several natural product and lipid databases. Screening of compound libraries predicts targeting of NLRX1 by conjugated trienes, polyketides, prenol lipids, sterol lipids, and coenzyme A-containing fatty acids for activating the NLRX1 pathway. The ligands of NLRX1 were identified by docking punicic acid (PUA), eleostearic acid (ESA), and docosahexaenoic acid (DHA) to the C-terminal fragment of the human NLRX1 (cNLRX1). Their binding and that of positive control RNA to cNLRX1 were experimentally determined by surface plasmon resonance (SPR) spectroscopy. In addition, the ligand binding sites of cNLRX1 were predicted *in silico* and validated experimentally. Target mutagenesis studies demonstrate that mutation of 4 critical residues ASP677, PHE680, PHE681, and GLU684 to alanine resulted in diminished affinity of PUA, ESA, and DHA to NLRX1. Consistent with the regulatory actions of NLRX1 on the NF-κB pathway, treatment of bone marrow derived macrophages (BMDM)s with PUA and DHA suppressed NF-κB activity in a NLRX1 dependent mechanism. In addition, a series of pre-clinical efficacy studies were performed using a mouse model of dextran sodium sulfate (DSS)-induced colitis. Our findings showed that the regulatory function of PUA on colitis is NLRX1 dependent. Thus, we identified novel small molecules that bind to NLRX1 and exert anti-inflammatory actions.

## Introduction

Nucleotide-binding domain and leucine-rich repeat containing (NLR) is a family of cytosolic pattern recognition receptors involved in innate immunity of plants and animals [1–6]. Three well-characterized NLR sub-groups (NLRP1, NLRP3 and NLRC4) could form a multi-protein complex termed the inflammasome [7]. NLR inflammasomes assemble in response to environmental stimuli (including dietary components), cell damage, microbiota translocation, and pathogen exposure [8–10]. NLR inflammasome formation results in the activation of Caspase 1, which has long been associated with IL-1β and IL-18 maturation and a unique form of cell death termed pyroptosis [11]. Mounting evidence demonstrates that NLRs play important roles in diverse inflammatory settings, including immune-mediated, infectious, and chronic inflammatory diseases [12–14]. More than 24 NLR proteins have been identified in humans and mice, but most remain uncharacterized [15, 16]. Hence, NLR regulation and activation remains poorly understood and the majority of their ligands remain unknown.

A partial structure of the NLR containing X1 (NLRX1) has recently been determined by X-ray crystallography [17]. NLRX1 is unique among the NLR family due to its intracellular localization on the mitochondria and its ability to negatively regulate type I interferon signaling and inflammatory cytokine responses [18, 19]. Mitochondria play vital roles in the energy metabolism of cells, reactive oxygen species (ROS) generation, programmed cell death, autophagy and innate antiviral responses [20]. NLRX1 functions as an *in vivo* checkpoint of IFN-I and IL-6 responses creating in a linkage between NLRX1, TRAF6 and NF-κB signaling [21]. *Nlrx1-/-* mice have increased morbidity and mortality in response to both LPS challenge and following virus infection [22]. Increased morbidity and mortality in these animals are associated with a failure to resolve dysregulated or excessive immune responses following pathogen clearance [23]. NLRX1 has also been shown to promote autophagy through direct interactions with the mitochondrial Tu translation elongation factor (TUFM) resulting in attenuation of ROS production [24]. NLRX1 is also required for ROS induction in response to pathogens [16, 25].

Computational modeling represents a cost-effective and efficient approach in therapeutic and nutraceutical discovery [26, 27]. Molecular docking approaches have been widely used in discovery of ligands and prediction of ligand-binding sites for proteins/receptors, such as TLR2 [28], TLR4 [29], and TLR5 [30]. The basic procedure of molecular docking is to sample binding geometry for compounds from large libraries into the structure of receptor targets by using molecular modeling approaches. Each compound is sampled in thousands to millions of possible poses and scored on the basis of its complementarity to the receptor [31]. Of the hundreds of thousands of molecules in the library, a selected number of top-scoring predicted ligands are subsequently tested for activity in experimental assays.

The solved three-dimensional structure of cNLRX1 was used for the presented *in silico* molecular docking studies [17]. RNA-seq data from colons of wild-type and *NLRX1-/-* mice with colitis demonstrates that NLRX1 regulates the expression of genes associated with lipid metabolism, thereby suggesting a link between lipid molecules and NLRX1 activity. The potential ligand binding domain of cNLRX1 was characterized for binding specificity by *in silico* docking of a variety of compounds from three libraries. Their respective binding to cNLRX1 was experimentally validated by surface plasmon resonance (SPR) spectroscopy along with several other compounds including ssRNA. Moreover, this predicted ligand-binding domain of cNLRX1 was experimentally validated by site-directed mutagenesis to be involved in punicic acid (PUA), eleostearic acid (ESA), and docosahexaenoic acid (DHA) binding. Since only RNA has been characterized to bind to NLRX1 to date, we identified novel NLRX1 ligands through virtual screening given the potential value of NLRX1 as a therapeutic target. The results of virtual screening highlight potential roles of NLRX1 targeting by plant-derived conjugated triene lipids, polyketides, prenol lipids, sterol lipids, and coenzyme A-containing fatty acids. In addition, we utilized molecular docking approaches to investigate the structure as well as the ligand binding domain of NLRX1. Following *in silico* studies, a series of biochemistry, *in vitro*, and *in vivo* experiments were performed to validate *in silico* predictions. PUA, ESA, and DHA were approved to bind with NLRX1. PUA and DHA exert anti-inflammatory efficacy in a NLRX1-dependent mechanism.

## Methods

### Molecule structure of NLRX1 and ligands

The crystal structure of the C-terminal fragment (residues 629–975) of the human NLRX1 (PDB ID: 3UN9) was downloaded from Protein Data Bank (PDB) at a 2.65 angstrom resolution [17]. The three-dimensional structures of compounds in the NIMML natural products database, including PUA,ESA, and DHA were obtained from PubChem [32], a database of chemical molecules maintained by the National Center for Biotechnology Information (NCBI, http://www.ncbi.nlm.nih.gov/). The PubChem IDs of PUA and ESA are 5281126 and 5281115, respectively. Both PUA and ESA have C_18_H_30_O_2_ as their molecular formula. PUA constitutes 64–83 percent of the pomegranate seed oil (PSO) [33], while ESA is at concentrations of 60–80 percent in tung and bitter gourd seed oils [34]. The molecular formula of DHA is C_28_H_33_O_2_ that can be obtained directly from maternal milk or fish oil [35].

### Molecular docking

AutoDock Vina is a state of the art software suite capable of high-throughput parallel computing for the assessment of potential NLR-phytochemical binding [36]. The software first computes the forces of free energy associated with the bound complex and subsequently evaluates the conformational space available for the complex formation between the target and the ligand. These methods are stochastic in nature, therefore they require repeated independent screens to exhaustively search all parameter spaces and provide confidence for the predictions. The docking studies were performed using the AutoDock Vina (version 1.1.2). AutoGrid, was used to define the search space, including grid box center and x,y,z-dimensions, while AutoDockTools was utilized as a front end interface and visualization [37]. Five bound conformations were generated for each compound. The docking was applied to the whole protein target, with a grid covering the whole surface of the protein. To search the entire surface of NLRX1, grid maps were set with the maximum spacing between grid points. The grid was a rectangular cuboid (67 Å × 100 Å × 100 Å) with grid points separated by 1.000 Å and centered at the middle of the protein. This grid was large enough to cover the entire surface of NLRX1 structure. The search for the optimum way to fit each compound into NLRX1 resulted in docking log files that contained records of docking, including binding energy of each predicted binding mode for all the compounds.

### Expression of recombinant cNLRX1 in *Pichia pastoris*

cNLRX1 (AA 667–970) was cloned into pPinkHC-Alpha-SDK, propagated in *E*.*coli* and ectopically integrated at multiple sites into *Pichia pastoris* (Strain 4: *ade2*, *prb1*, *pep4*). White colonies on drop adenine media were screened for protein expression via small-scale induction and both pellets and supernatants were screened for expression via western blot using an anti-polyhistidine (6x) antibody (Immunology Consultants Laboratory, Inc). A single transformant with high levels of expression was chosen for further studies. Cultures were grown at 30°C for 3 days at 240 RPM in 1 L BMSY media (1% yeast extract, 2% peptone, 100 mM potassium phosphate buffer pH 6.0, 1.34% yeast nitrogen base, 0.0004% biotin, 1% sorbitol) using a 2L baffled flask with a mesh top. Cells were harvest at 10,000 x g for 15 min at room temperature and re-suspended in 500 mL of Pichia induction media (100 mM potassium phosphate buffer pH 6.0, 1.34% yeast nitrogen base, 0.0004% biotin, 1% sorbitol, 1% methanol) in a mesh top 1L baffled flask. Every 24 hours post induction cells were fed 10 mL of methanol and 10% filter sterilized sorbitol for 4 days post induction. Cells were subsequently harvested at 10,000 x g for 15 min and were either used immediately or stored at -80°C.

### Purification of recombinant cNLRX1

Cells were re-suspended in 40 mL of ice-cold lysis buffer (50mM Sodium Phosphate, 200mM sodium chloride, 25mM Sucrose, 0.1% T-100, pH = 7.2) and sonicated 8 times at max amplitude using a 15 sec ON 45 sec OFF cycle. Lysed cells were centrifuged at 15,000 x g for 30 min at 4°C. Supernatant were loaded onto a 150 mL Super-loop and purified using a programmed immobilized metal affinity chromatography gradient protocol on the AKTA prime plus. Bound protein was washed with 40 column volumes of wash buffer (50 mM sodium phosphate buffer, 300 mM sodium chloride, 20 mM imidazole, pH 8.0) and eluted in 1 mL fractions over a 0.02-1M imidazole 40 mL gradient. Purity was assessed via SDS-PAGE. Proteins were further purified via gel filtration chromoatograhy using a Superdex75 column (16/600) in 50 mM sodium phosphate, 300 mM sodium chloride pH 8.0. Protein concentration was determined via absorbance at 280 nm. Protein was dialyzed (1:1000) twice at 4°C for a minimum of 8 hours in appropriate buffers for downstream assays.

### Sensor chip preparation

Direct binding experiments were performed via the Biacore T200 Surface Plasmon Resonance (SPR) Technology (Georgetown University). The flow rates were 10 μL/min for all capture and initial testing studies and 100 μL/min for affinity studies. The cNLRX1 was immobilized to a CM4 or CM5 chip by amine coupling method. Two adjacent surfaces were activated by injection of a 1:1 (v:v) mixture of 0.1 M N-Hydroxysuccinimide (NHS) and 0.4 M 1-Ethyl-3-(3-dimethyl-aminopropyl)-carbodiimide hydrochloride (EDC) for 720 seconds. Experimental flow cell was then injected with the cNLRX1 that was diluted in 10 mM pH 4.0 sodium acetate buffer to a final concentration of 25 μg/mL. Both experimental and the reference flow cells were inactivated by injection of 1M Ethanolamine-HCl pH 8.0 for 720 seconds.

### Kinetic studies

A final cNLRX1 surface density (RL) of 13100 RU equivalents to an Rmax of 48 RU was reached. Binding affinity was calculated following injection of small molecules at 7 different concentrations (ranging from 40 μM to 1 μM) in triplicate. Binding affinity was determined following dose dependent injections of ssRNA (8 μM, 4 μM, 2 μM, 1 μM, and 0.5 uM) in triplicates. Negative control compound, 1,14-bis (3,b-dimethoxypheny1)-tetradecane, was tested at a concentration of 20 μM. Injection time was 60 seconds and dissociation time was 300 seconds. Binding studies involving PUA, ESA, and RNA were conducted in 25 mM MOPS (pH 6.5), 150 mM NaCl, 0.05% Tween-20, 5% DMSO. Binding studies incolving DHA were conducted in HBS-P (0.01 M HEPES pH 7.4, 0.15 M NaCl, 0.005% v/v Surfactant P2). Data was analyzed by using the BiaEvaluation software (GE Healthcare) with 1:1 binding model for steady state affinity. Raw data was exported and graphed using Prizm for Mac v5.0d and excel.

### Mutation analysis of binding site

Based on molecular docking results, the potential binding site of NLRX1 was identified. AutoDockTools were used to measure the distance between the PUA and NLRX1. Those residues of NLRX1 with a distance less than 5 Å were selected as predicted amino acids in the potential binding site. To verify the prediction of binding site by molecular docking and understand different roles of residues in the binding site, mutation analysis was performed by producing a mutant of cNLRX1. The predicted residues within the ligand binding domain of cNLRX1 were mutated to alanine. Kinetic studies were performed on the mutant using same approaches described above.

### NF-κB ELISA

Bone marrow derived macrophages (BMDM)s were isolated from hind legs of wild type and *Nlrx1-/-* mice in sterile conditions and cultured for 7 days as described [38]. After stimulated with LPS (1μg/ml) for 12 hours, cells were treated with control (medium), 40 μM PUA, and 40 μM DHA for 12 hours. Nuclear extraction was performed on colon homogenates using the Active Motif Nuclear Extraction Kit (Carlsbad, CA) as previously described [39]. ELISA was performed on nuclear fractions using the Active Motif TransAM^®^ NF-κB p65 kit according to the manufacturer’s instructions.

### Animal procedures

C57BL/6J wild-type (WT) and *Nlrx1-/-* mice (n = 10) ranging from 8–10 weeks of age [38, 40] were administered 8% DSS (average 500,000 molecular weight) in drinking water for six days. Control mice received tap water. Mice were randomly allotted into groups treated additionally with either PUA (40 mg/kg body weight in 0.2 mL) or PBS, as a control, via oral gavage. On day 6, mice were euthanized via carbon dioxide narcosis with secondary cervical dislocation. All mice were weighed and scored daily. Clinical scores were based on physical appearance (0–3), fecal consistency (0–3), presence of rectal bleeding (0–4), and weight loss (0–3) and assigned a compounded score for overall disease activity (0–4).

### Gene expression

Total RNA was isolated from whole mouse colon using a Qiagen (Germantown, MD) RNA isolation mini kit. Complementary DNA (cDNA) was generated from each sample (1 μg RNA) using the iScript cDNA synthesis kit (Bio-Rad). Standards were produced through a polymerase chain reaction on the cDNA with Taq DNA polymerase from Invitrogen. The amplicon was purified using the Mini-Elute PCR purification kit from Qiagen. Expression levels were obtained through quantitative real-time PCR on a Bio-Rad (Hercules, CA) CFX 96 Thermal Cycler using the Bio-Rad SYBR Green Supermix. For analysis, the starting amount of anti-microbial peptide cDNA was compared to that of beta-actin, as a control. cDNA concentrations for genes of interest were examined by RT-PCR as described [39].

### Histopathology

Colonic samples were fixed in 10% buffered formalin, embedded in paraffin, processed routinely and sectioned at 5 μm. Sections were stained with hematoxylin and eosin (H&E) and then examined and graded using an Olympus microscope (Olympus America, Center Valley, PA) and ImagePro software. Sections were graded for leukocytic infiltration (0–4), epithelial erosion (0–4) and mucosal thickening (0–4).

### Analysis of an RNA-seq dataset encompassing NLRX1 knockout and wild type mice challenged with a dextran sodium sulfate (DSS) model of inflammatory bowel disease (IBD)

Total RNA was isolated from colons of WT control, *NLRX1-/-* control, WT DSS, and *NLRX1-/-* DSS on day 7 of challenge. Each experimental grouping consisted of three replicates. RNA was assessed using Illumina Hiseq for whole transcriptome gene expression at Virginia Bioinformatics Institute Core Lab Facilities. High quality reads were mapped to RefSeq (mm10 from http://genome.ucsc.edu/) using Bowtie (version: 1.0.0) with parameters set to ‘-l 25-I 1-X 1000-a-m 200’. RPKM (reads per kilobase per million) values were used to measure expression level. Data was imported into Ingenuity Pathway Analysis (IPA) to search for functional differences. Hierarchical based clustering was used to group significant (FDR p-value < 0.05) genes via the hclust method in R (version 3.1.3). Combinatorial fold changes (*Nlrx1-/-* non-DSS to WT non-DSS, *Nlrx1-/-* DSS to *Nlrx1-/-* non-DSS, WT DSS to WT non-DSS, *Nlrx1-/-* DSS to WT DSS) were first calculated prior to clustering. The combinatorial fold changes were clustered initially into four groups. Each group of genes was further clustered into sub-groups. Gene Ontology mapping was also used to identify shared functionality within clusters of genes with similar patterns across experiments groupings.

### Ethics statement

All experimental procedures were approved by the Virginia Tech Institutional Animal Care and Use Committee (IACUC) under protocol 14-013-VBI and met or exceeded requirements of the Public Health Service/National Institutes of Health and the Animal Welfare Act.

### Statistics

Data were analyzed as a completely randomized design. To determine the statistical significance of the model, analysis of variance (ANOVA) was performed using the general linear model procedure of R, and probability value (P) <0.05 was considered to be significant. When the model was significant, ANOVA was followed by multiple comparison method to identify pairwise treatments with significant difference.

## Results

### Structure assessment

Two levels of assessment were performed to determine the quality of the structure of the C-terminal fragment of the human NLRX1 (PDB ID: 3UN9). The atomic empirical mean force potential ANOLEA was used to assess packing quality of the structure. ANOLEA performs energy calculations on a protein chain, evaluating the “non-local environment” (NLE) of each heavy atom in the molecule. In the ANOLEA plot, the y-axis of the plot represents the energy for each amino acid of the protein chain. Negative energy values (in green) represent a favorable energy environment whereas positive values (in red), an unfavorable energy environment for a given amino acid. Using this strategy, 95.2% of amino acid residues in the cNLRX1 structure appeared in a favorable environment (S1A Fig). The PROCHECK suite of programs assesses the stereochemical quality of a given protein structure. The Ramachandran plot from PROCHECK also corroborates the quality of the structure, with 89.1% *phi* and *psi* angles in the favored core region, 10.5% in allowed regions, and only 0.4% in disallowed regions (S1B Fig).

### *In silico* and *in vitro* Binding of ligands to cNLRX1

**Fig 1:**
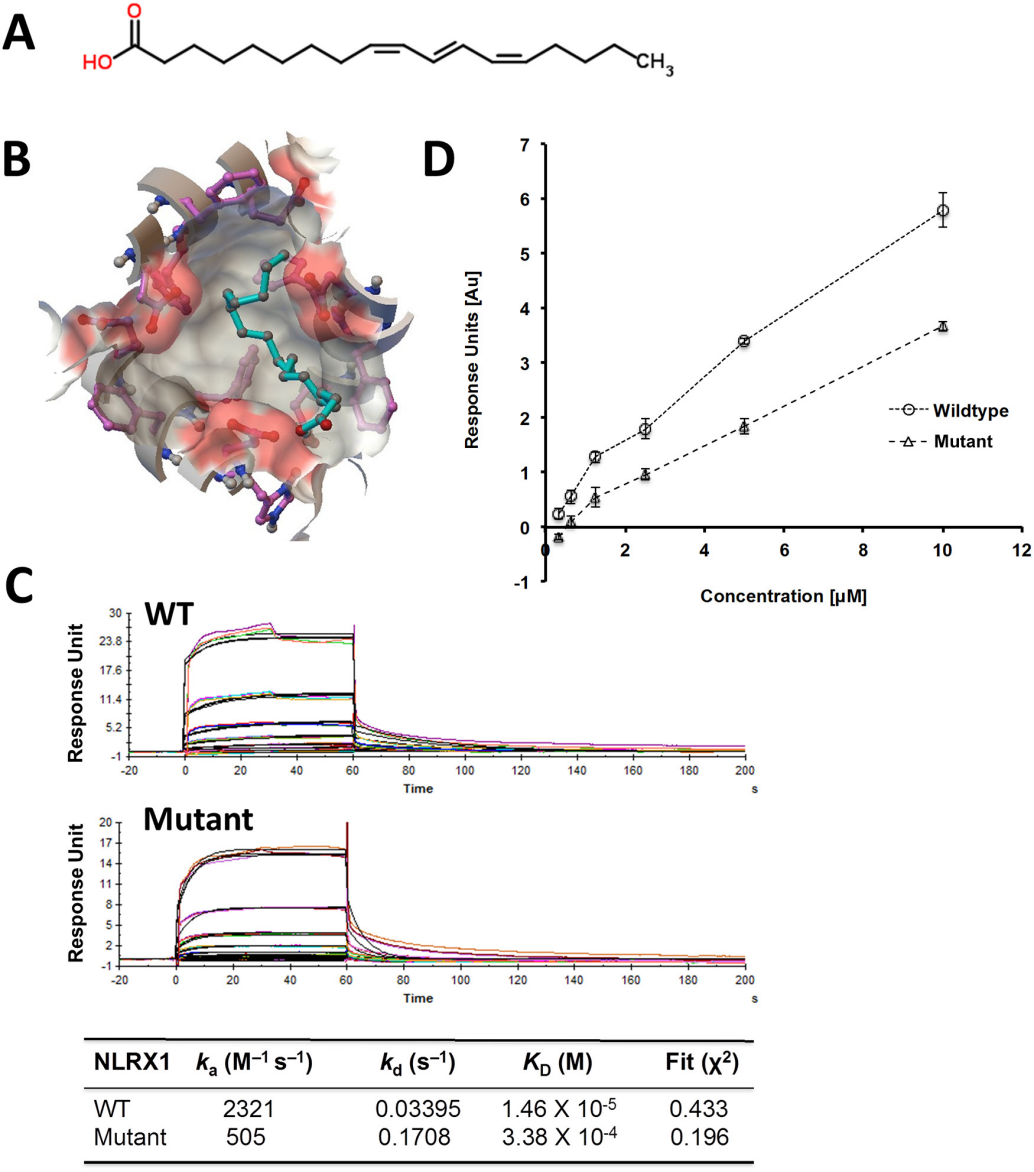
Binding of punicic acid (PUA) with cNLRX1 wildtype and mutant. A) Chemical structure of PUA. B) Interactions between PUA and cNLRX1 predicted by molecular docking. PUA is in cyan ball-and-stick representation surrounded by molecular surface of the binding site with coloring by element. The free energy of binding is -6.2 kcal/mol. C) SPR sensograms for the binding of varying concentrations of PUA (40.0, 20.0, 10.0, 5.0, 2.5, 1.25 and 0.0 μM) to immobilized cNLRX1 wildtype (WT) and ASP677, PHE680, PHE681, and GLU684 to alanine mutant (Mutant). The equilibrium constant of dissociation, K_D_, of PUA is 1.46 × 10^−5^ M for WT and 3.38 × 10^−4^ M for Mutant. D) Strength of association plot showing maximum response units for captured cNLRX1 WT or Mutant for a given concentration of PUA

**Fig 2:**
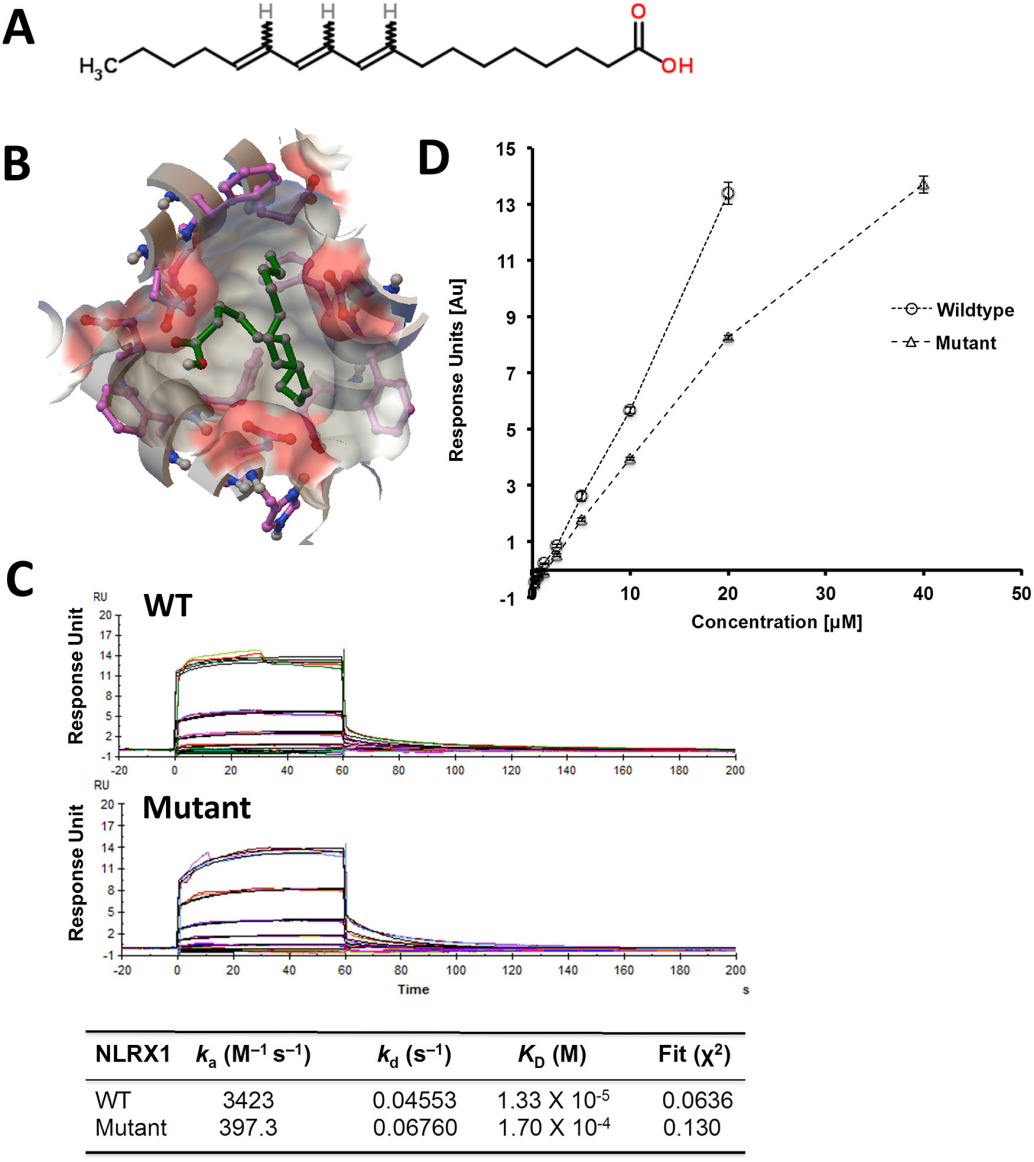
Binding of eleostearic acid (ESA) with cNLRX1 wildtype and mutant. A) Chemical structure of ESA. B) Interactions between ESA and NLRX1 predicted by molecular docking. ESA is in green ball-and-stick representation surrounded by molecular surface of the binding site with coloring by element. The free energy of binding is -6.2 kcal/mol. C) SPR sensograms for the binding of varying concentrations of ESA (40.0, 20.0, 10.0, 5.0, 2.5, 1.25 and 0.0 μM) to immobilized cNLRX1 (WT) and ASP677, PHE680, PHE681, and GLU684 to alanine mutant (Mutant). The equilibrium constant of dissociation, K_D_, of ESA is 1.33 × 10^−5^ M for WT and 1.70 × 10^−4^ M for Mutant. D) Strength of association plot showing maximum response units for captured cNLRX1 WT or Mutant for a given concentration of ESA.

We determined the predicted binding energies for PUA and ESA with cNLRX1 respectively using molecular docking approaches. The dockings of PUA and ESA into the crystal structure of cNLRX1 was performed with AutoDock Vina (Version 1.1.2). The docking was applied to the whole protein target, with a grid covering the entire surface of the protein. The predicted free energy of binding of PUA and ESA to NLRX1 was determined to be -6.2 kcal/mol (Figs 1 and 2). Purified cNLRX1 was determined to be structured and migrated as a tight multimeric complex by CD Spectroscopy and gel filtration chromatography respectively (S2 Fig). Predicted binding was validated by direct binding experiments performed via SPR spectrocopy. The equilibrium constant of dissociation (K_D_) of PUA and ESA with cNLRX1 (wild type) is 1.46×10^−5^ M (Fig 1C) and 1.33×10^−5^ M (Fig 2C). cNLRX1 also bound ssRNA in a dose dependent manner (S3A Fig). Further, we identified a negative control compounds, 1,14-bis (3,b-dimethoxypheny1)-tetradecane, based on our *in silico* prediction (free energy of binding: -4.9 kcal/mol) and determined via SPR spectroscopy showing that the negative control failed to bind cNLRX1 (S3B Fig).

### Characterization of ligand binding domain of cNLRX1

In addition to calculating the binding energetics between cNLRX1 and a variety of ligands, a putative ligand binding domain (LBD) for cNLRX1 was identified using AutoDockTools. A spherical region, with a radius of 5 Å, was selected centered on PUA docked onto cNLRX1. The residues of cNLRX1 within this spherical region were considered to have the highest probability in the PUA interaction and compromised the LBD. This domain includes ASP677, PHE680, PHE681, and GLU684, which fall within the leucine-rich repeats of cNLRX1. To determine the role of the putative LBD, we mutated ASP677, PHE680, PHE681, and GLU684 to alanine (Mutant cNLRX1). Both PUA and ESA bound Mutant cNLRX1 at approximately an order of magnitude lower than the wild type form (K_D_ 3.38×10^−4^ M (Fig 1C) and 1.70×10^−4^ M (Fig 2C) respectively) suggesting these predicted residues play a role in binding PUA and ESA. Binding specificity was further determined as cNLRX1 bound ssRNA in a dose dependent manner (S3A Fig), but not 1,14-bis (3,b-dimethoxypheny1)-tetradecane (S3B Fig).

### Analysis of virtual screening results

**Fig 3:**
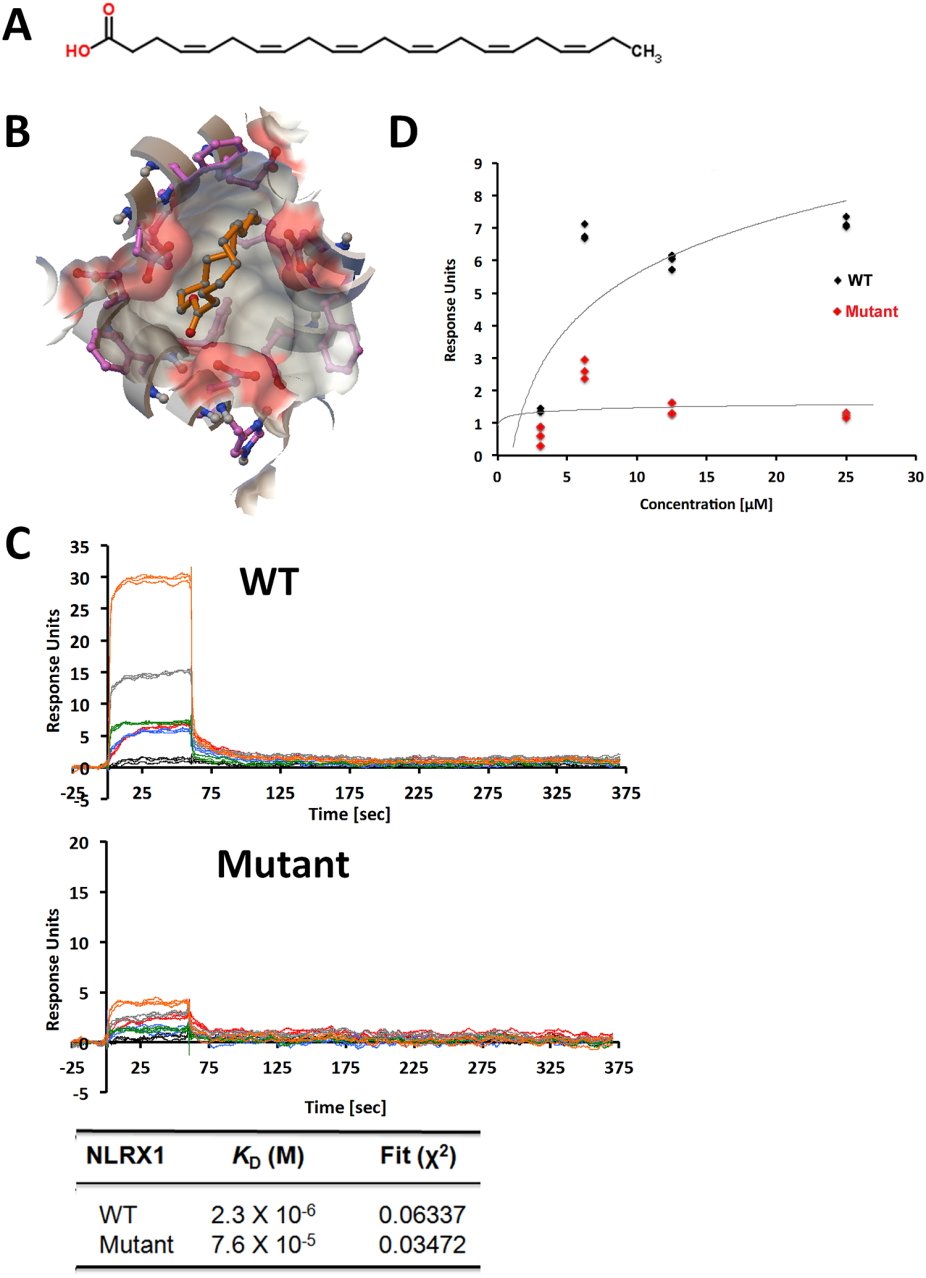
Binding of docosahexaenoic acid (DHA) with cNLRX1 wildtype and mutant. A) Chemical structure of DHA. B) Interactions between DHA and cNLRX1 predicted by molecular docking. DHA is in orange ball-and-stick representation surrounded by molecular surface of the binding site with coloring by element. The free energy of binding is -8.0 kcal/mol. C) SPR sensograms for the binding of varying concentrations of DHA (100.0, 50.0, 25.0, 12.5, 6.23, and 3.125 μM) to immobilized cNLRX1 wild-type (WT) and ASP677, PHE680, PHE681, and GLU684 to alanine mutant (Mutant). The equilibrium constant of dissociation, KD, of DHA is 2.3 × 10–6 M for WT and 75.9 × 10–6 M for Mutant. D) Strength of association plot showing maximum response units for captured cNLRX1 WT or Mutant for a given concentration of DHA.

LMSD contains structures and annotations of biologically relevant lipids. It is a relational database, in which all lipids have been classified, named and drawn according to the comprehensive classification, nomenclature and drawing system. Since the goal of our studies is to identify which classes of lipids have highest possibilities to interact with NLRX1, 1,000 lipids were randomly selected from the whole database to represent all eight classes of lipids. Free energy of binding was calculated by AutoDock Vina for each lipid to NLRX1. The latter represents the sum of the total intermolecular energy, total internal energy and torsional free energy minus the energy of the unbound system. Low free energy of binding indicates high binding affinity. S4 Fig shows the average free energy of binding of different classes of lipids to NLRX1. This analysis indicates that polyketides, prenol lipids, and sterol lipids have higher average binding affinities with NLRX1 compared with other classes of lipids. S1 and S2 Tables lists the free energy of binding of the top ranked and bottom ranked lipids. The clusters of lipids that have stronger binding affinity are listed as potential candidates. In addition, the lipids that are clustered with very low binding affinity (listed at the bottom of the S1 and S2 Tables) could serve as negative control. Taxa-4(5),11(12)-diene (LMPR0104390002) was predicted to have strongest interactions with NLRX1 within all sampled lipids, whose free energy of binding is -10.6 kcal/mol. DHA is a highly ranked interactor identified by virtual screening of LMSD and it is also commercially available natural product. DHA bound cNLRX1 with a K_D_ of 2.3×10^−6^ M via SPR spectroscopy (Fig 3). Additionally, top ranked NLRX1 binding natural products were also identified through screening of ZINC database and shown in S2 Table. ZINC12655082 was predicted to have strongest interactions with NLRX1, whose free energy of binding is -13.2 kcal/mol.

### Ability of NLRX1 ligands to down-modulate NF-κB activity

**Fig 4:**
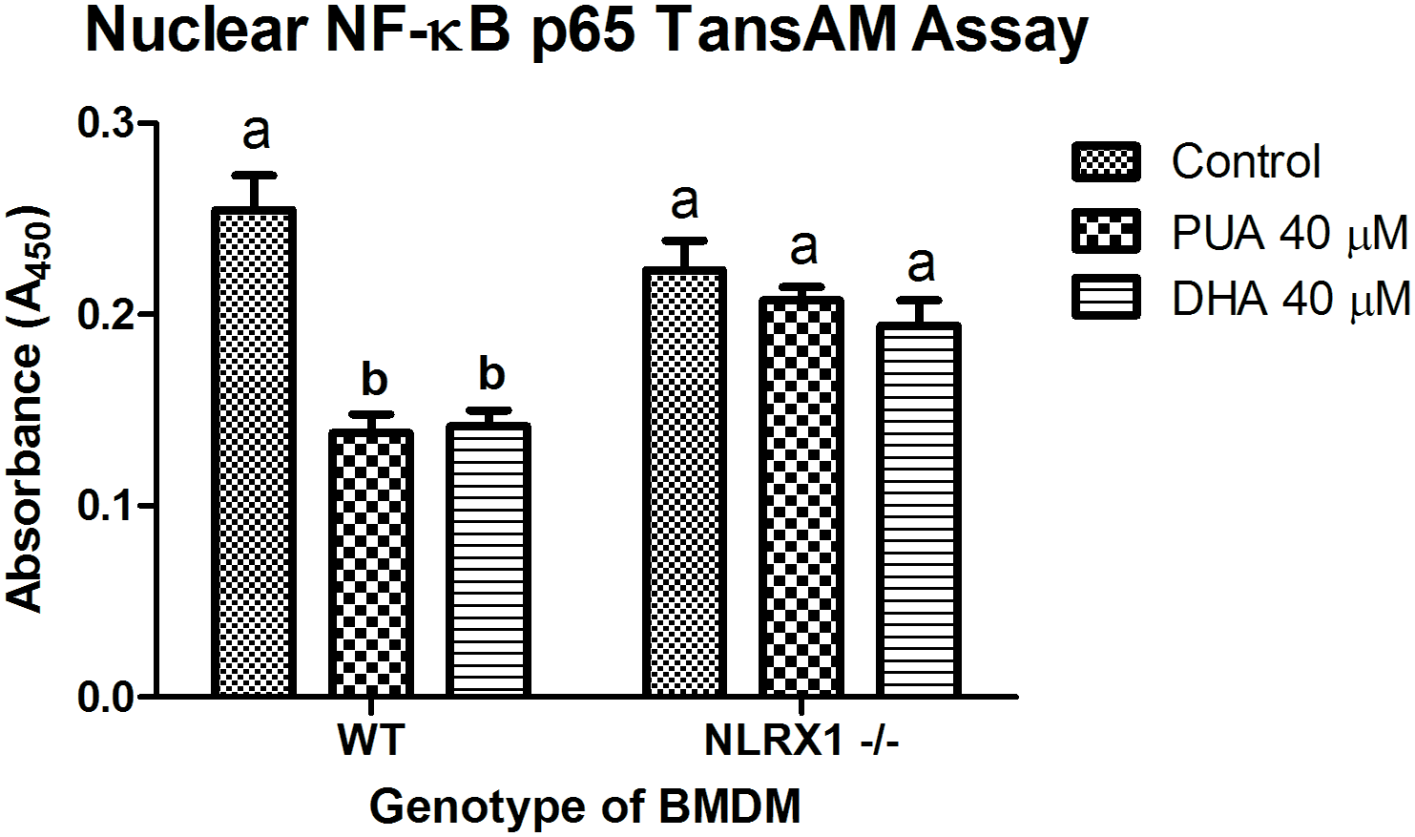
Effect of NLRX1 disruption on NF-κB activation in Bone marrow derived macrophage (BMDM). BMDMs were isolated from hind legs obtained fromm wild type and *Nlrx1-/-* in sterile conditions and cultured for 7 days After stimulated with LPS (1μg/ml) for 12 hours, cells were treated with control (medium), 40 μM PUA, and 40 μM DHA for 12 hours. Nuclear extraction was performed on colon homogenates using the Active Motif Nuclear Extraction Kit (Carlsbad, CA). ELISA was performed on both cytoplasmic and nuclear fractions using the Active Motif TransAM^®^ NF-κB p65 kit according to the manufacturer’s instructions. Letter superscripts indicate significant (P-value < 0.05) differences by ANOVA.

The NF-κB activation by NLRX1 ligands was detected and quantified by using the Trans-AM^™^ NF-κB p65 ELISA-based assay (Active Motif, Carlsbad, CA) as described in the manual of the Active Motif TransAM^®^ NF-κB p65 kit. Our results show that NLRX1 ligands, PUA and DHA, suppressed the NF-κB activity in BMDMs with LPS stimulation and the deficiency of *Nlrx1* abrogates the effect of PUA and DHA (Fig 4).

### Regulation of colitis by PUA via a NLRX1 dependent mechanism

**Fig 5:**
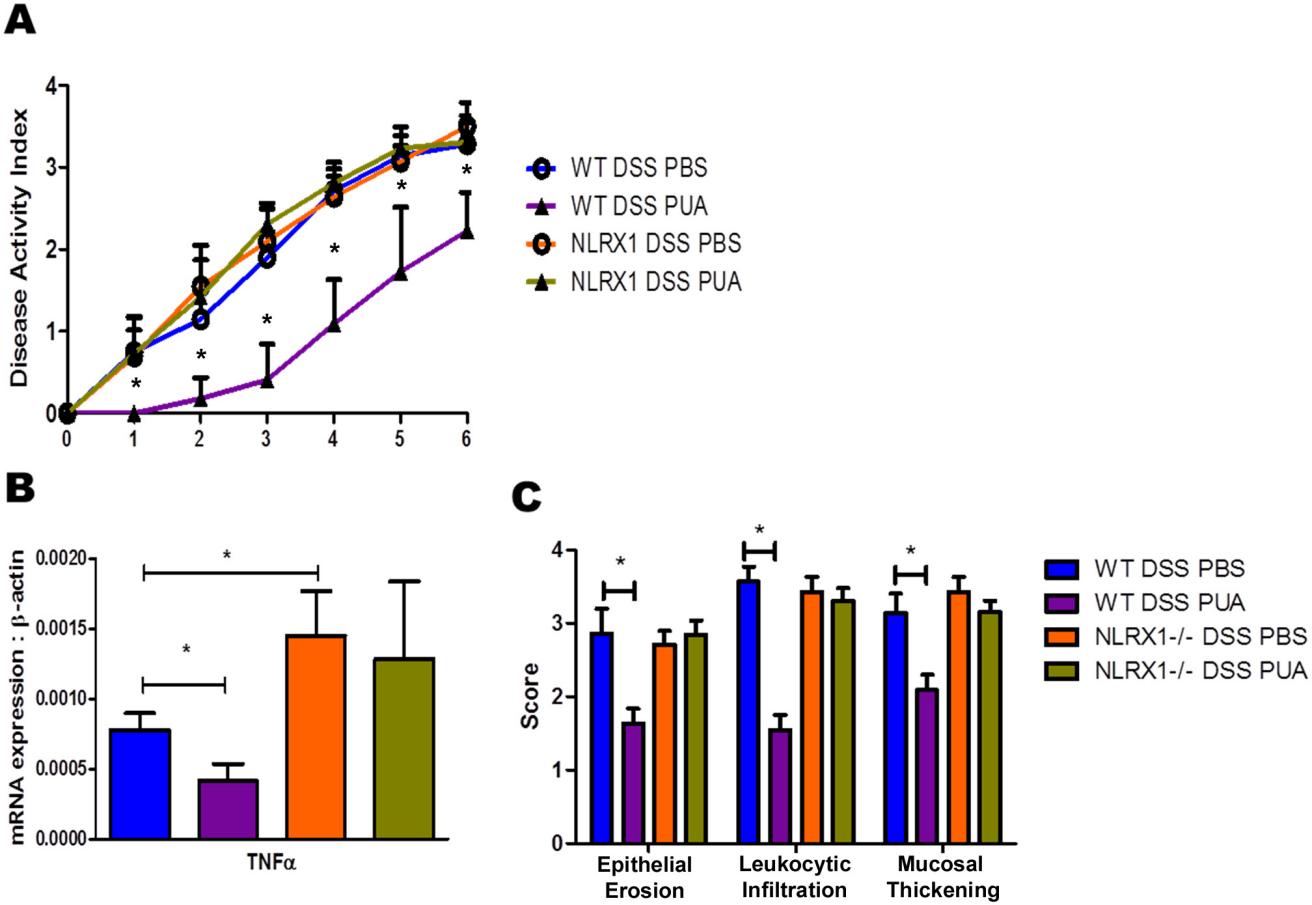
Effect of PUA on WT and *Nlrx1-/-* mice in DSS-induced colitis (10 mice per group). A) Average disease activity of treatment groups scored 0–4 daily for six days. B) mRNA expression of TNFα in whole colon on day six of DSS treatment. C) Histopathological assessment of colons by epithelial erosion (EE), leukocytic infiltration (LI), and mucosal thickening (MT). Asterisk indicates P-value < 0.05 by ANOVA.

To characterize the selectivity of the anti-inflammatory mechanism of PUA in the gut mucosa as NLRX1-dependent, WT mice and *Nlrx1-/-* mice were challenged with six days of DSS-induced colitis and treated with either PUA or PBS, as a control, daily via oral gavage. Mice were observed daily for disease activity with WT mice given PUA showing significant symptom regulation, while *Nlrx1-/-* counterparts had no observable inhibition of colitis (Fig 5A). To further investigate the effect of PUA on colitis, colons were removed and processed on day six for gene expression by qRT-PCR and assessed for histopathological differences. Indeed, the colons reflected the symptomatic separation between genotypes when administered PUA. WT mice treated with PUA had significantly lower detectable tumor necrosis factor alpha (TNFα) than controls, demonstrating a far greater inflammatory response on the molecular level, while *Nlrx1-/-* mice were overall unaffected (Fig 5B). In line with these results were the pathological differences observed in the colon histology. *Nlrx1-/-* colons failed to show any inflammatory amelioration in the cells from PUA treatment, while WT mice had significantly lower scores across the board in epithelial erosion, leukocytic infiltration, and mucosal thickening (Fig 5C). These in-vivo results present ample evidence for the interaction of NLRX1 and PUA to modulate mucosal immune responses in the gut and relieve inflammation.

### Expression of fatty acid metabolism related genes altered by absence of NLRX1

**Fig 6:**
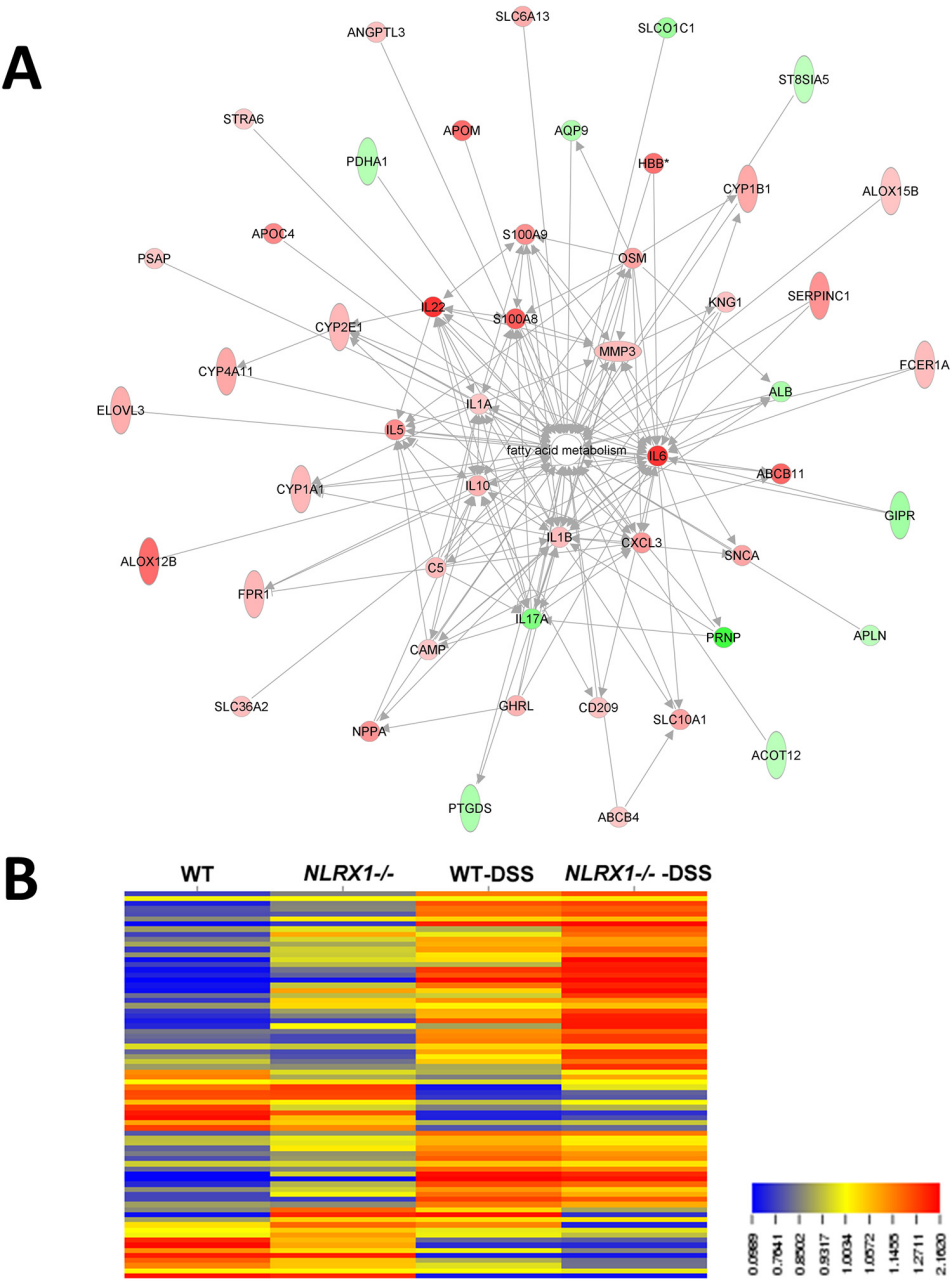
Expression of fatty acid metabolism related genes altered by absence of NLRX1. A) RNA-seq expression data was gathered after 7 day exposure to DSS in wild-type and *Nlrx1-/-* mice. Interactions of genes significantly altered in comparison of *Nlrx1-/-* treated with DSS to wild-type treated with DSS. Red indicates an up-regulation and green indicates a down-regulation. Image was generated using Ingenuity Pathway Analysis. B) Expression profile of genes encoding for enzymes and transporters in fatty acid metabolism pathways.

Through analysis of an RNA-seq dataset encompassing *Nlrx1-/-* and wild type mice challenged with a dextran sodium sulfate (DSS) model of colitis, we observed a significant dysregulation of lipid metabolism associated genes (Fig 6). Out of the 971 genes significantly altered by the genotype and treatment interaction, 54 were identified to have a connection to fatty acid metabolism by Ingenuity Pathway Analysis. Within altered genes in the fatty acid metabolism function, there exists an additional division. Genes most often connected more closely to immunological mechanisms, cytokines, chemokines and other secreted factors, tended to be more highly connected with other genes and generally increased with *Nlrx1-/-* mice (centrally located within Fig 6A). However, the genes more directly connected to fatty acid metabolism, the lipid transporters and metabolic enzymes, experienced a mixed effect and displayed both up and down regulation within *Nlrx1-/-* mice, suggesting an altered ability of the cell to process lipids. Following hierarchical clustering of significant genes, four out of the sixteen clusters displayed large amounts of genes within lipid metabolism. Most strikingly, cluster A, a cluster marked by a general linear increase from left to right across groups, contained 31 genes within lipid metabolic process (GO:0006629) out of the total 76 genes within the cluster, amounting to an enrichment greater than 5 and a p-value of 1.04E-19. The other three clusters of genes with large lipid and fatty acid memberships notably had decreased expression of genes within *Nlrx1-/-* compared to WT both with DSS and without DSS.

## Discussion

The NLRs are a family of novel cytosolic pattern recognition receptors conserved across plants and animals and associated with non-self and modified-self recognition [2–5, 15]. NLR dysregulation has been associated with a wide variety of infectious and immune-mediated diseases, including inflammatory bowel disease (IBD) [41–44]. For instance, NOD1 and NOD2 are best characterized in IBD, with various mutations reported to impact pathology and disease [45–49]. NLRX1 is a mitochondrial-associated protein involved in down-regulating inflammation following viral exposure [50]. Indeed, NLRX1 regulates host responses to single-stranded RNA viruses [51] and interferon production [52]. Additionally, NLRX1 is a regulator of inflammation, autophagy, and ROS production in response to viral and bacterial pathogens ranging from *Chlamydia* [25] to influenza [51, 53]. NLRX1 contains an N-terminal mitochondria-targeting sequence required for its trafficking to the mitochondrial membrane. Mechanistically, NLRX1 was shown to down-regulate mitochondrial anti-viral signaling protein (MAVS)-mediated type I interferon production, interfere with the TLR-TRAF6-NF-κB pathways, and enhance virus induced-autophagy. However, NLRX1 is also involved in the generation of ROS induced by TNFα and *Shigella* infection magnifying the JNK and NF-κB signaling. The precise role of NLRX1 remains controversial and further research is required to validate its pro or anti-inflammatory properties [41]. Together, these findings suggest a role for NLRX1 as a therapeutic target for autoimmune and inflammatory diseases.

Combined with the differences in colonic pathology between the two genotypes, the changes in these lipid metabolism pathways, specifically those of the inflammatory signaling eicosanoid pathway, may be connected to the absence of NLRX1. This could suggest that NLRX1 may act as a cytosolic sensor of dysregulated lipid/metabolite profiles and may contribute to the regulatory control of the pathway. Indeed, our global transcriptomic analyses on RNA from colons of WT and *NLRX1-/-* mice is consistent with this hypothesis since the loss of NLRX1 resulted in a significant dysregulation of lipid metabolism-associated genes. Therefore, the aim of this study was to identify novel classes of NLRX1 ligands (lipids and natural products) based on the three-dimensional structural of cNLRX1.

In the present work, molecules in three compound libraries were docked to the structure of cNLRX1 using the blind docking method. We firstly identified PUA, ESA, and DHA as ligands of NLRX1 in molecular modeling studies, and subsequently their respective binding to NLRX1 was validated experimentally. In addition, the ligand binding domain of cNLRX1 was identified and key residues within the ligand binding domain were predicted by evaluation on effects of amino acid substitutions on protein function. Furthermore, the ability of PUA and DHA to down-modulate NF-κB activity is examined by performing NF-κB ELISA, which showed that both PUA and DHA suppressed the NF-κB activity in a NLRX1 dependent mechanism. The NLRX1 dependent anti-inflammatory efficacy of PUA was further validated in the DSS-induced colitis model.

In addition, the analysis of virtual screening results indicated that polyketides, prenol lipids, and sterol lipids have higher average binding affinities with NLRX1 compared with other classes of lipids in LMSD. Polyketides are a class of secondary metabolites produced by animal, plant, bacterial, fungal and marine sources in order to impart to them some survival advantage, which have been commonly used as anti-microbial, anti-parasitic, and anti-cancer agents, such as erythromycins, tetracyclines, avermectins, and antitumor epothilones [54]. Prenol lipids are synthesized from the five-carbon-unit precursors isopentenyl diphosphate and dimethylallyl diphosphate [55]. Simple prenol lipids, such as carotenoids, function as antioxidants and as precursors of vitamin A [56]. Plant sterol lipids are naturally occurring food components which are well known for their serum low-density lipoprotein (LDL)-cholesterol-lowering ability. Recent research indications that plant sterol lipids might enhance immune function and have anti-inflammatory effects [57]. In addition, our virtual screening shows that coenzyme A (CoA) containing lipids (fatty acyl CoAs), such as 6Z,9Z,12Z,15Z,18Z-tetracosapentaenoyl-CoA and 1-(3-hydroxy-6Z,9Z,12Z,15Z,18Z,21Z-tetracosahexaenoyl)-CoA also bind to NLRX1. CoA is involved in the synthesis and oxidation of lipids, and CoA-containing lipids are implicated in mitochondrial energy metabolism [58, 59], which indicates that NLRX1 may also play important roles in mitochondrial energy metabolism. This function of NLRX1 is consistent with its intracellular localization as a mitochondrial associated protein. On the basis of these findings, we propose that the function of NLRX1 may be regulated by interaction with conjugated trienes, polyketides, prenol lipids, sterol lipids, and coenzyme A-containing fatty acids, and play important roles in the interface of immunity and metabolism.

Polypharmacology suggests that more effective drugs can be developed by specifically modulating multiple targets. Considering that complex diseases may require complex therapeutic approaches, a drug that hits multiple sensitive nodes belonging to a network of interacting targets may offer the potential for higher efficacy and limited drawbacks generally arising from the use of a single-target drug or a combination of multiple drugs [60]. PUA is a conjugated triene fatty acid naturally found at high concentrations in the seed of *Punica granatum* [61] and *Trichosanthes kirilowii* [62]. PUA constitutes 64% to 83% of the pomegranate seed oil, which also contains minor amounts of ESA, catalpic acid (CAA) and jacaric acid (JAA). All these acids are collectively known as conjugated linolenic acids (CLnAs) that are stereo- or regioisomers of each other. PUA has been shown to robustly bind and activate PPARγ. Also, increasing PPARγ-responsive gene expression was shown to improve diabetes and gut inflammation [63]. The loss of PPARγ in immune cells impaired their ability to modulate glucose homeostasis and obesity-related inflammation [64]. PUA was also identified to ameliorate DSS-induced colitis and spontaneous pan-enteritis in *IL-10−/−* mice [65]. The loss of PPARγ in immune cells impaired the ability of PUA to ameliorate experimental colitis, strongly suggesting that PUA could modulate mucosal immune responses and therefore ameliorate gut inflammation through a PPARγ-dependent mechanism, which antagonizes NF-κB, STAT and AP-1 [66]. Similarly, ESA was also able to regulate gut inflammation. However, immune modulatory actions of ESA may be both PPARγ dependent and PPARγ independent [63]. Combined with previous discovery, our results show that PUA and ESA can bind to both PPARγ and NLRX1, two intracellular regulatory molecules that are activated by a broad array of lipids, which may provide a novel and more efficient treatment against gut inflammation by modulating immunity and metabolism.

In conclusion, this study employed an integrated drug discovery pipeline consisting of molecular docking approaches followed by biochemical, *in vitro* and *in vivo* validation in mice with colitis. We performed large-scale screening of compound libraries based on the crystal structure of NLRX1 and identified novel putative naturally occurring compounds for the treatment of inflammation. Our studies indicate that NLRX1 acts as a cytosolic sensor of lipids that may operate in the interface of immunity and metabolism, and identified the binding site of these lipid molecules to NLRX1. This knowledge is a first step towards accelerating the development of novel NLRX1-based therapeutics for autoimmune and inflammatory diseases.

## Supporting Information

S1 Fig ANOLEA plot and Ramachandran plot of the cNLRX1 structure. A) In the ANOLEA plot, negative values (green) indicate residues in a favorable environment and positive values (red) indicate residues in an unfavorable environment. B) In the Ramachandran plot, the favored and most favored region is represented by yellow and red respectively; pale yellow represents the generously allowed, and disallowed regions are in white.
(DOCX)

S2 Fig Assessment of Secondary Structure and Oligomeric State of purified cNLRX1 wildtype and mutant protein. A) Mean residual ellipticity of NLRX1 Wildtype (NLRX1) and Mutant (Mut) for the Far-UV region as determined by Circular Dichroism Spectroscopy at 25°C. B) Photon Multiplier Tube voltage (HT voltage) of NLRX1 and Mutant. C) Predicted secondary structure of NLRX1 and Mutant by the CONTINLL algorithm. D) Gel filtration chromatography of purified NLRX1, Mutant, and blue dextran using a Superdex 75 column (16/600) at 1 ml/min in 50 mM sodium phosphate 300 mM sodium chloride, pH8.
(DOCX)

S3 Fig Determination of NLRX1 binding affinity to control ssRNA and Natural Products by SPR Spectroscopy. A) Binding of varying concentrations of ssRNA (8 μM, 4 μM, 2 μM, 1 μM, and 0.5 uM) with captured cNLRX1. The equilibrium dissociation constant, K_D_, is 1.326 × 10^−5^ M. B) Binding of punicic Acid (PUA, -6.2 kcal/mol free energy of binding), eleostearic acid (ESA,-6.2 kcal/mol free energy of binding), and 1,14-bis (3,b-dimethoxypheny1)-tetradecane (-4.8 kcal/mol free energy of binding) with cNLRX1. Ligands were injected at a concentration of 20 μM. No binding was observed for 1,14-bis (3,b-dimethoxypheny1)-tetradecane. Kinetics for the interaction of PUA and ESA with cNLRX1 are presented in Figs 3 and 4 respectively.
(DOCX)

S4 Fig Average free energy of binding of different classes of lipids to cNLRX1. LMFA: fatty acyls, LMGL: glycerolipids, LMGP: glycerophospholipids, LMPK: polyketides, LMPR: prenol lipids, LMSL: sterol lipids, LMSP: sphingolipids, LMST: sterol lipids. All the structures of lipids were obtained from LIPID MAPS Structure Database (LMSD). The free energy of binding was calculated by using AutoDock Vina (version 1.1.2).
(DOCX)

S1 Table Free energy of binding of top and bottom ranked lipids to cNLRX1 with the respective structures and common names. (LMFA: fatty acyls, LMGL: glycerolipids, LMGP: glycerophospholipids, LMPK: polyketides, LMPR: prenol lipids, LMSL: sterol lipids, LMSP: sphingolipids, LMST: sterol lipids).
(DOCX)

S2 Table Free energy of binding of top 33 and bottom 36 ranked lipids to cNLRX1. (LMFA: fatty acyls, LMGL: glycerolipids, LMGP: glycerophospholipids, LMPK: polyketides, LMPR: prenol lipids, LMSL: sterol lipids, LMSP: sphingolipids, LMST: sterol lipids).
(DOCX)
