# Supplementary material for: Modeling-Enabled Characterization of Novel NLRX1 Ligands
Source: PLoS One. 2015 Dec 29;10(12):e0145420. doi: 10.1371/journal.pone.0145420 (PMC4694766; doi:10.1371/journal.pone.0145420)
Supplement: S1 Fig — A) In the ANOLEA plot, negative values (green) indicate residues in a favorable environment and positive values (red) indicate residues in an unfavorable environment. B) In the Ramachandran plot, the favored and most favored region is represented by yellow and red respectively; pale yellow represents the generously allowed, and disallowed regions are in white. (DOCX) [file pone.0145420.s001.docx]

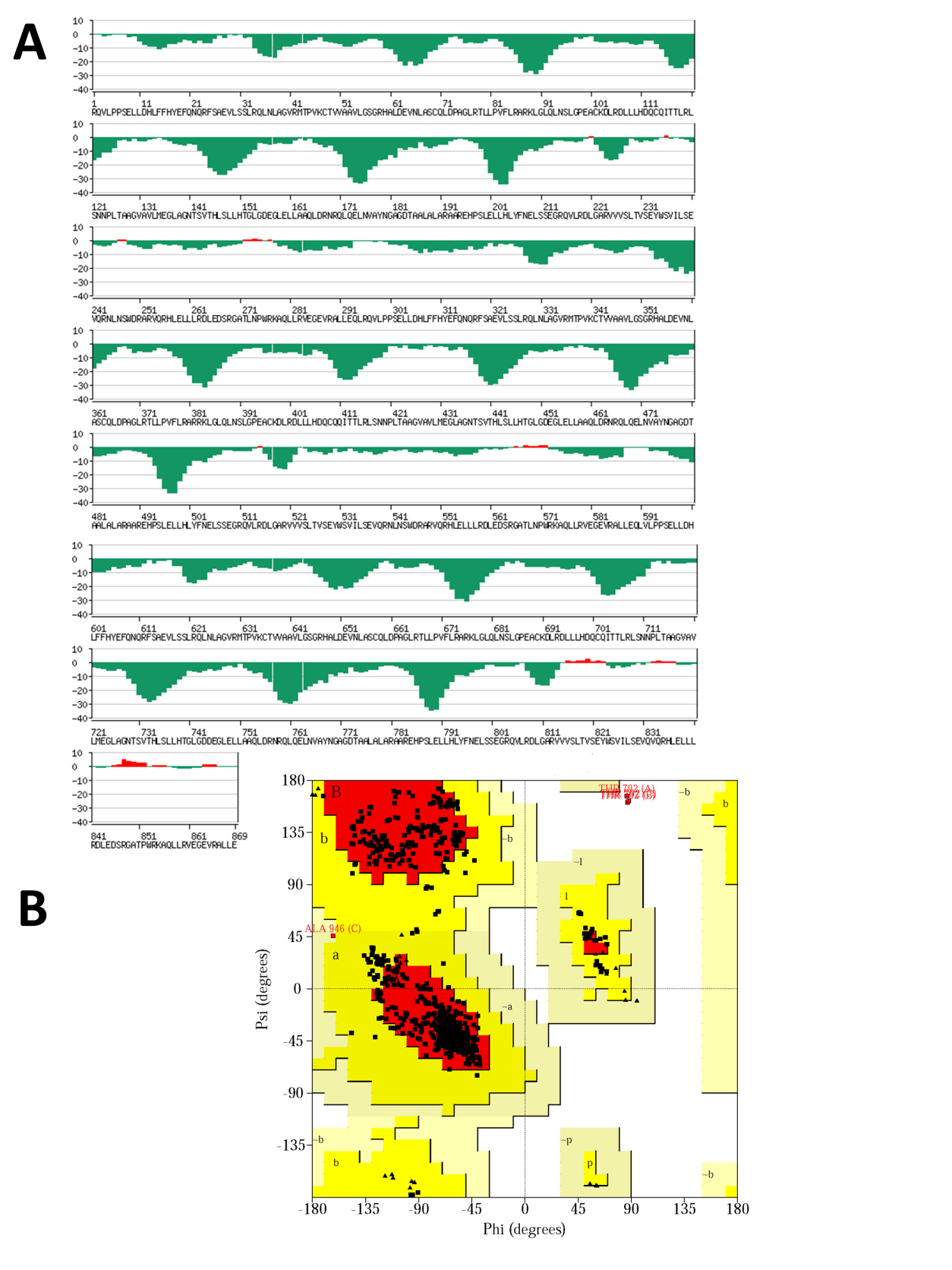


**S1 Fig.** **ANOLEA plot and Ramachandran plot of the cNLRX1 structure.** A) In the ANOLEA plot, negative values (green) indicate residues in a favorable environment and positive values (red) indicate residues in an unfavorable environment. B) In the Ramachandran plot, the favored and most favored region is represented by yellow and red respectively; pale yellow represents the generously allowed, and disallowed regions are in white.
