# Supplementary material for: Modeling-Enabled Characterization of Novel NLRX1 Ligands
Source: PLoS One. 2015 Dec 29;10(12):e0145420. doi: 10.1371/journal.pone.0145420 (PMC4694766; doi:10.1371/journal.pone.0145420)
Supplement: S2 Fig — A) Mean residual ellipticity of NLRX1 Wildtype (NLRX1) and Mutant (Mut) for the Far-UV region as determined by Circular Dichroism Spectroscopy at 25°C. B) Photon Multiplier Tube voltage (HT voltage) of NLRX1 and Mutant. C) Predicted secondary structure of NLRX1 and Mutant by the CONTINLL algorithm. D) Gel filtration chromatography of purified NLRX1, Mutant, and blue dextran using a Superdex 75 column (16/600) at 1 ml/min in 50 mM sodium phosphate 300 mM sodium chloride, pH8. (DOCX) [file pone.0145420.s002.docx]

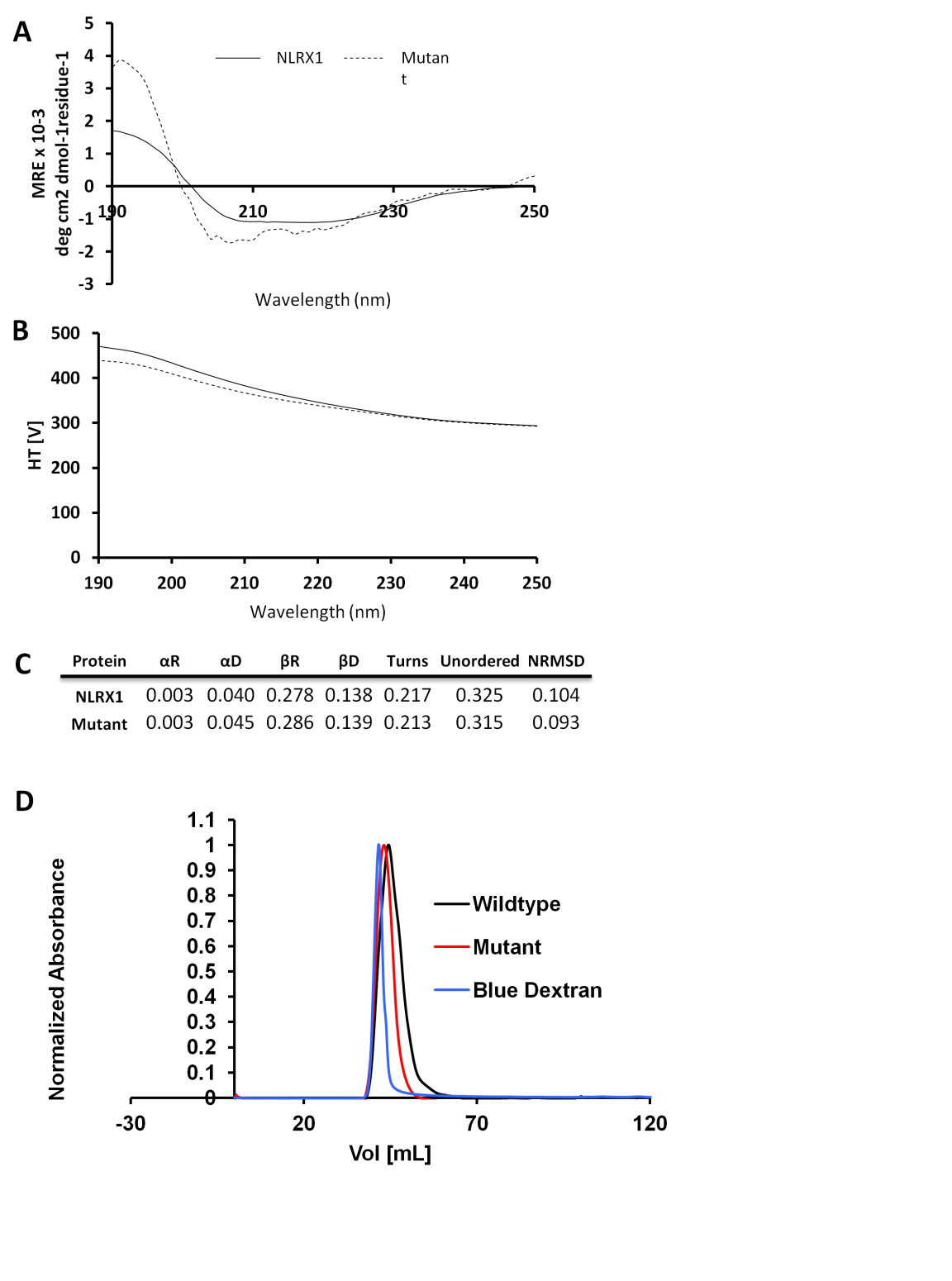


**S2 Fig.** **Assessment of Secondary Structure and Oligomeric State of purified NLRX1 wildtype and mutant protein.** A) Mean residualof NLRX1 wildtype (NLRX1) and mutant (NLRX1m1) for the Far-UV region as determined by Circular Dichroism Spectroscopy at 25°C. B) Photon Multiplier Tube voltage (HT voltage) of NLRX1 and NLRX1M1. C) Predicted secondary structure of NLRX1 and NLRX1M1 by the CONTINLL algorithm. D) Gel filtration chromatography of purified NLRX1, NLRX1M1, and blue dextran using a Superdex 75 column (16/600) at 1 ml/min in 50 mM sodium phosphate 300 mM sodium chloride, pH8.
