# Supplementary material for: Modeling-Enabled Characterization of Novel NLRX1 Ligands
Source: PLoS One. 2015 Dec 29;10(12):e0145420. doi: 10.1371/journal.pone.0145420 (PMC4694766; doi:10.1371/journal.pone.0145420)
Supplement: S3 Fig — A) Binding of varying concentrations of ssRNA (8 μM, 4 μM, 2 μM, 1 μM, and 0.5 uM) with captured cNLRX1. The equilibrium dissociation constant, KD, is 1.326 × 10^−5^ M. B) Binding of punicic Acid (PUA, -6.2 kcal/mol free energy of binding), eleostearic acid (ESA,-6.2 kcal/mol free energy of binding), and 1,14-bis (3,b-dimethoxypheny1)-tetradecane (-4.8 kcal/mol free energy of binding) with cNLRX1. Ligands were injected at a concentration of 20 μM. No binding was observed for 1,14-bis (3,b-dimethoxypheny1)-tetradecane. Kinetics for the interaction of PUA and ESA with cNLRX1 are presented in Figs 3 and 4 respectively. (DOCX) [file pone.0145420.s003.docx]

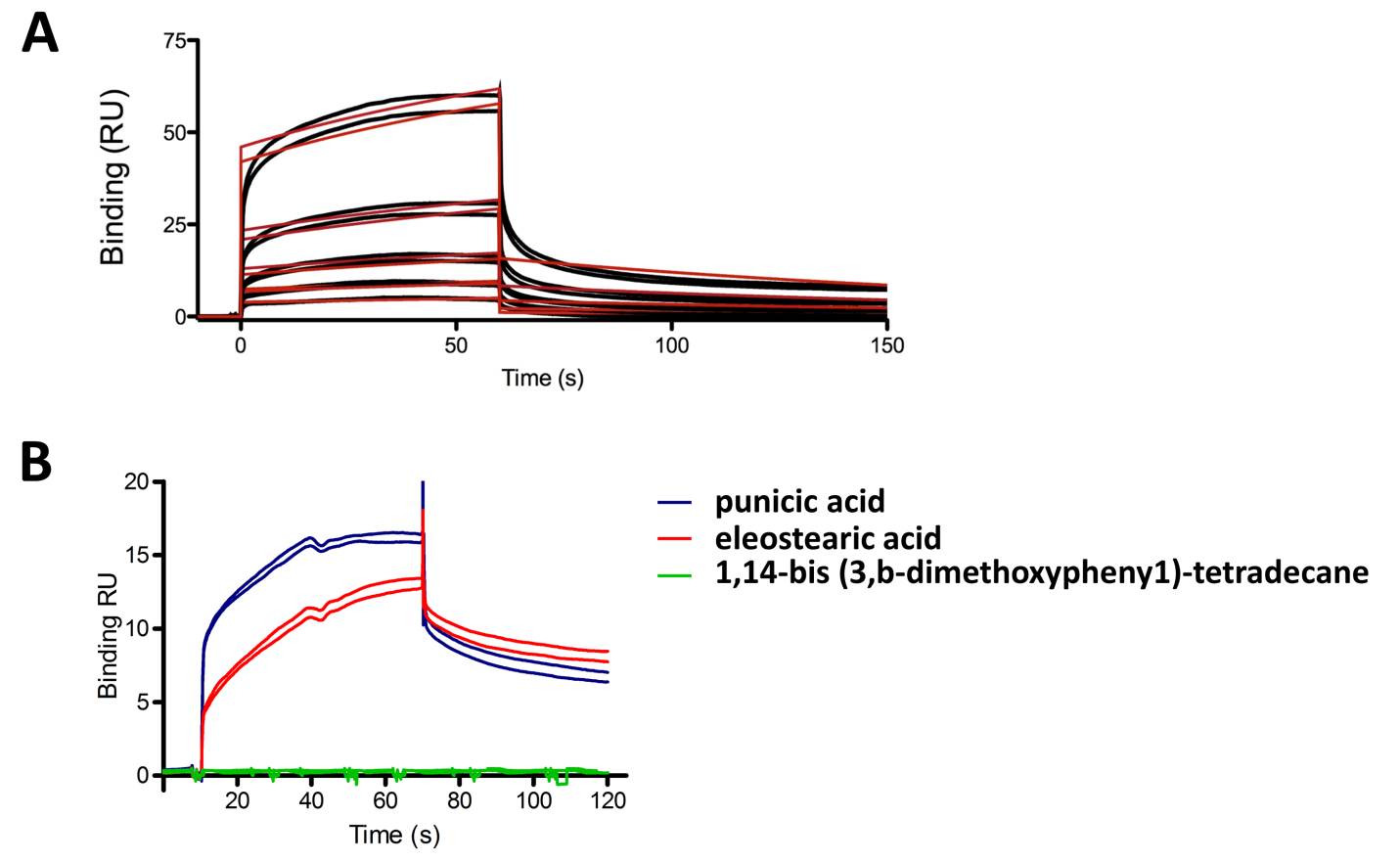


**S3 Fig.** **Determination of NLRX1 binding affinity to control ssRNA and Natural Products by SPR Spectroscopy**. A) Binding of varying concentrations of ssRNA (8 μM, 4 μM, 2 μM, 1 μM, and 0.5 uM) with captured cNLRX1. The equilibrium dissociation constant, K_D_, is 1.326 × 10^-5^ M. B) Binding of punicic Acid (PUA, -6.2 kcal/mol free energy of binding), eleostearic acid (ESA,-6.2 kcal/mol free energy of binding), and 1,14-bis (3,b-dimethoxypheny1)-tetradecane (-4.8 kcal/mol free energy of binding) with cNLRX1. Ligands were injected at a concentration of 20 μM. No binding was observed for 1,14-bis (3,b-dimethoxypheny1)-tetradecane. Kinetics for the interaction of PUA and ESA with cNLRX1 are presented in Fig. 3 and Fig. 4 respectively.
