# Supplementary material for: Modeling-Enabled Characterization of Novel NLRX1 Ligands
Source: PLoS One. 2015 Dec 29;10(12):e0145420. doi: 10.1371/journal.pone.0145420 (PMC4694766; doi:10.1371/journal.pone.0145420)
Supplement: S4 Fig — LMFA: fatty acyls, LMGL: glycerolipids, LMGP: glycerophospholipids, LMPK: polyketides, LMPR: prenol lipids, LMSL: sterol lipids, LMSP: sphingolipids, LMST: sterol lipids. All the structures of lipids were obtained from LIPID MAPS Structure Database (LMSD). The free energy of binding was calculated by using AutoDock Vina (version 1.1.2). (DOCX) [file pone.0145420.s004.docx]

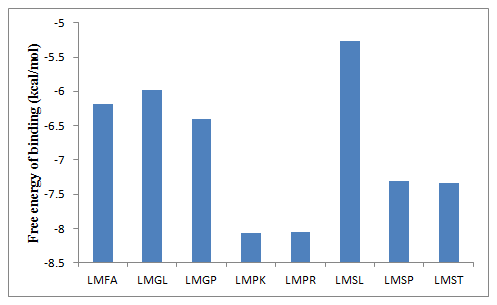


**S4 Fig.** **Average free energy of binding of different classes of lipids to cNLRX1.** LMFA: fatty acyls, LMGL: glycerolipids, LMGP: glycerophospholipids, LMPK: polyketides, LMPR: prenol lipids, LMSL: sterol lipids, LMSP: sphingolipids, LMST: sterol lipids. All the structures of lipids were obtained from LIPID MAPS Structure Database (LMSD). The free energy of binding was calculated by using AutoDock Vina (version 1.1.2).
