# Supplementary material for: Modeling-Enabled Characterization of Novel NLRX1 Ligands
Source: PLoS One. 2015 Dec 29;10(12):e0145420. doi: 10.1371/journal.pone.0145420 (PMC4694766; doi:10.1371/journal.pone.0145420)
Supplement: S1 Table — (LMFA: fatty acyls, LMGL: glycerolipids, LMGP: glycerophospholipids, LMPK: polyketides, LMPR: prenol lipids, LMSL: sterol lipids, LMSP: sphingolipids, LMST: sterol lipids). (DOCX) [file pone.0145420.s005.docx]

**S1 Table. Free energy of binding of top and bottom ranked lipids to cNLRX1** **with the respective structures and common names** (LMFA: fatty acyls, LMGL: glycerolipids, LMGP: glycerophospholipids, LMPK: polyketides, LMPR: prenol lipids, LMSL: sterol lipids, LMSP: sphingolipids, LMST: sterol lipids)**.**

| **Lipid ID in LipidMaps** | **Structure** | **Common/Systematic Name** | **Free energy of binding (kcal/mol)** |
| --- | --- | --- | --- |
| LMPR0104390002 | 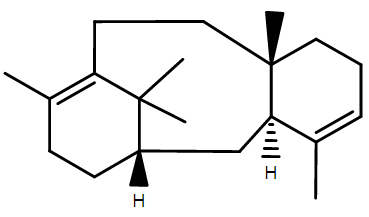 | Taxa-4(5),11(12)-diene | -10.6 |
| LMPK12120523 | 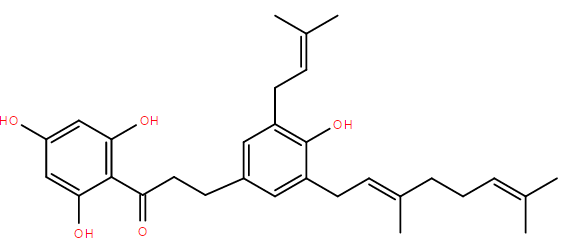 | 3-Geranyl-4,2',4',6'-tetrahydroxy-5-prenyldihydrochalcone | -10.2 |
| LMPK12140517 | 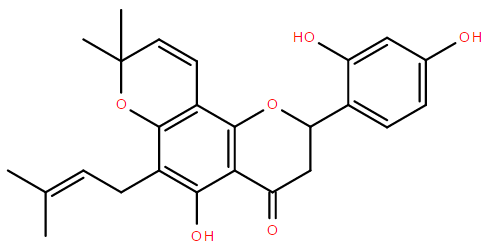 | Euchrenone a9 | -10.1 |
| LMST02020000 | 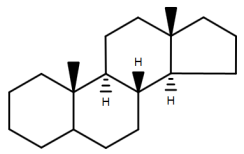 | Androstrane skeleton | -10.1 |
| LMST02020084 | 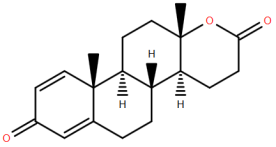 | Testolactone | -10.1 |
| LMFA05000110 | 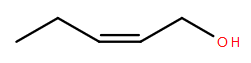 | 2Z-Penten-1-ol | -4.3 |
| LMFA11000342 | 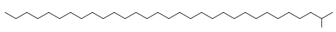 | 2-Methylhentriacontane | -4.3 |
| LMGP01020024 | 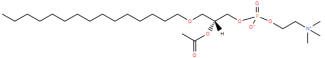 | PC(O-15:0/2:0) | -4.3 |
| LMFA11000471 | 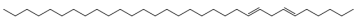 | 6,9-Hentriacontadiene | -4.2 |
| LMSL03000982 | 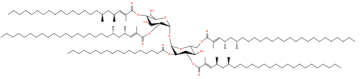 | PAT18(25:1(2E)(2Me,4Me[S],6Me[S])/26:1(2E)(2Me,4Me[S],6Me[S])/25:1(2E)(2Me,4Me[S],6Me[S])/22:1(2E)(2Me,4Me[S],6Me[S])) | -4 |
