# Supplementary material for: Modeling-Enabled Characterization of Novel NLRX1 Ligands
Source: PLoS One. 2015 Dec 29;10(12):e0145420. doi: 10.1371/journal.pone.0145420 (PMC4694766; doi:10.1371/journal.pone.0145420)
Supplement: S2 Table — (LMFA: fatty acyls, LMGL: glycerolipids, LMGP: glycerophospholipids, LMPK: polyketides, LMPR: prenol lipids, LMSL: sterol lipids, LMSP: sphingolipids, LMST: sterol lipids). (DOCX) [file pone.0145420.s006.docx]

**S2 Table.** **Free energy of binding of top 33 and bottom 36 ranked lipids to cNLRX1** (LMFA: fatty acyls, LMGL: glycerolipids, LMGP: glycerophospholipids, LMPK: polyketides, LMPR: prenol lipids, LMSL: sterol lipids, LMSP: sphingolipids, LMST: sterol lipids).

| **Lipid ID in LipidMaps** | **Free energy of binding (kcal/mol)** |
| --- | --- |
| LMPR0104390002 | -10.6 |
| LMPK12120523 | -10.2 |
| LMPK12140517; LMST02020000; LMST02020084 | -10.1 |
| LMPK12111515 | -10 |
| LMPK12140170; LMPR0104350002; LMST03020646 | -9.8 |
| LMPK12010166; LMST03020085 | -9.7 |
| LMPK12111194; LMPK12140519; LMST04010264 | -9.6 |
| LMPK12140307; LMST03020021; LMST03020142 | -9.5 |
| LMPK12080022; LMST03020350 | -9.4 |
| LMPK12110171; LMPR0104190002; LMST03020124 | -9.3 |
| LMPK12060011; LMPK12120011 | -9.2 |
| LMPK12020226; LMPK12030015; LMPK12060073; LMPK12111686;  LMPK12140139 | -9.1 |
| LMPK12110572; LMPK12111619; LMPR0102060013; LMST01160008 | -9 |
| LMFA02000003; LMFA02000074; LMFA07010448; LMFA07070001;  LMGL03011107; LMGL03011870;  LMSL03000532; LMSL03001124;  LMSL03001182 | -5 |
| LMFA01020042;LMFA07060006;  LMSL03000298;LMSL03001147 | -4.9 |
| LMFA07060018; LMFA11000097;  LMSL03000158; LMSL03000209;  LMSL03000633; LMSL03001011;  LMSL03001233 | -4.8 |
| LMFA07010219; LMGL03016194;  LMSL03000556; LMSL03000745;  LMSL03001239; LMSP03010078 | -4.7 |
| LMFA01030352; LMFA12000081 | -4.6 |
| LMFA01050077; LMFA07010170;  LMFA07010428 | -4.5 |
| LMFA05000110; LMFA11000342;  LMGP01020024 | -4.3 |
| LMFA11000471 | -4.2 |
| LMSL03000982 | -4 |
